# Structural and Functional MRI Differences in Master Sommeliers: A Pilot Study on Expertise in the Brain

**DOI:** 10.3389/fnhum.2016.00414

**Published:** 2016-08-22

**Authors:** Sarah J. Banks, Karthik R. Sreenivasan, David M. Weintraub, Deanna Baldock, Michael Noback, Meghan E. Pierce, Johannes Frasnelli, Jay James, Erik Beall, Xiaowei Zhuang, Dietmar Cordes, Gabriel C. Leger

**Affiliations:** ^1^Cleveland Clinic Lou Ruvo Center for Brain Health, Las VegasNV, USA; ^2^Université du Québec à Trois-Rivières, Trois-RivièresQC, Canada; ^3^Chappellet Vineyard, NapaCA, USA; ^4^Cleveland Clinic Radiology, ClevelandOH, USA

**Keywords:** olfaction, expertise, MRI, entorhinal cortex, insula

## Abstract

Our experiences, even as adults, shape our brains. Regional differences have been found in experts, with the regions associated with their particular skill-set. Functional differences have also been noted in brain activation patterns in some experts. This study uses multimodal techniques to assess structural and functional patterns that differ between experts and non-experts. Sommeliers are experts in wine and thus in olfaction. We assessed differences in Master Sommeliers’ brains, compared with controls, in structure and also in functional response to olfactory and visual judgment tasks. MRI data were analyzed using voxel-based morphometry as well as automated parcellation to assess structural properties, and group differences between tasks were calculated. Results indicate enhanced volume in the right insula and entorhinal cortex, with the cortical thickness of the entorhinal correlating with experience. There were regional activation differences in a large area involving the right olfactory and memory regions, with heightened activation specifically for sommeliers during an olfactory task. Our results indicate that sommeliers’ brains show specialization in the expected regions of the olfactory and memory networks, and also in regions important in integration of internal sensory stimuli and external cues. Overall, these differences suggest that specialized expertise and training might result in enhancements in the brain well into adulthood. This is particularly important given the regions involved, which are the first to be impacted by many neurodegenerative diseases.

## Introduction

Several studies have assessed the development of new skills in adulthood and associated changes in brain structure. Diverse areas of expertise have been studied including taxi driving and hippocampal volume (Maguire et al., 2000), juggling and visual and motor regions of the cortex (Draganski et al., 2004), musicians and the auditory cortex (Bermudez and Zatorre, 2005), and more recently expertise in perfume has been associated with olfactory regions of the frontal lobe (Delon-Martin et al., 2013). Similarly, there have been studies looking at distinct functional activation patterns in experts. For example, musicians show a distinct pattern of prefrontal activity compared with non-musicians when listening to different rhythms (Chen et al., 2008), perfumers show distinct patterns of activation in the olfactory regions and hippocampus when imaging smells (Plailly et al., 2012) and even sommeliers, or wine experts, showed enhanced regions of the memory network when tasting wines during an fMRI study (Pazart et al., 2014). Sommeliers’ brains are of particular interest since their expertise is centered on the chemo-senses, olfaction and gustation, but associated with multiple other functions including memory, judgment, and the amalgamation of this with other senses. The olfactory regions are relevant to diseases such as Alzheimer’s and Parkinson’s, where initial neurodegeneration is isolated to regions important in smell. Furthermore, given that sommeliers are experts not just in a single domain (e.g., olfaction and gustatory) but combine these, we felt that they may have specialization in regions important in integrating sensory information.

While several studies have assessed either structural or functional differences in experts, few look at both in the same cohort. It seems likely that there is a mechanistic relationship between functional and structural changes in brains as they gain expertise. In highly complex activities and areas of expertise that are multi-faceted in nature, regional structural differences may tell only part of the story. The same regions should show both functional and structural differences between experts and non-experts. Here, we investigate the differences between the brains of Master Sommeliers and non-wine-experts with an emphasis on multimodal analysis analyzing both structural and functional brain differences.

Sommeliers are experts in wine. Master Sommeliers have worked their way to the top of their field. In addition to depending heavily on their sense of smell and taste, sommeliers learn to draw on their memory and other senses, notably vision. Specifically, sommeliers use mental imagery, such as imagining the fruits and vegetables in a grocery store, when judging wine. This is part of the Deductive Tasting Method by which they are trained and which they use in their examinations, including the blind tasting component^1^. By the end of their training, they have accumulated a wealth of knowledge linked to the smell of the wines, which is always evaluated prior to tasting. Thus, we expected the sommeliers in our study to show enhanced size and activity of regions important in olfactory memory, multimodal integration, designation of hedonic value, and emotional salience. We further expected the differences in functional activation to be specific to olfactory-based tasks, not tasks in other sensory modalities.

Prior studies have pointed to the right hemisphere as being dominant for many aspects of olfaction, including olfactory memory (Jones-Gotman and Zatorre, 1993). Visual imagery has been shown to involve all aspects of the visual system, even primary regions (Klein et al., 2000; Slotnick et al., 2012). Integration of sensory and meta-cognitive data is frequently associated with the insula. The anterior insula bilaterally has been shown to be critical in olfactory and gustatory integration (Craig, 2011; Seubert et al., 2013; Small et al., 2013). It is also considered important in integrating sensory stimuli across modalities, and assigning hedonic value and emotional salience to stimuli [e.g., disgust (Calder et al., 2000)]. We thus expected sommeliers and non-expert controls to differ in activation of regions in the right hemisphere important in memory, olfaction, vision, as well as the insula. Perception, familiarity and labeling of odors have been shown to involve regions of the frontal lobe including the orbitofrontal regions and anterior cingulate (Royet et al., 1999; Gottfried and Dolan, 2003; de Araujo et al., 2005) so these regions may also be involved in the process under investigation.

There is limited literature on imaging of olfactory expertise. In a structural neuroimaging study, perfumers showed increases in gray-matter volume in the bilateral gyrus rectus, and the anterior piriform cortex, the latter correlating with experience (Delon-Martin et al., 2013). Functional neuroimaging studies have also been conducted in sommeliers while they tasted (as opposed to smelled) wine. The authors of one such study explored gustatory processing of wine compared with glucose in seven sommeliers and seven non-experts (Castriota-Scanderbeg et al., 2005), and found enhanced activation of the anterior part of the left insula, adjoining orbitofrontal cortex, as well as the dorsolateral prefrontal cortex bilaterally in sommeliers. In a similar study comparing 10 sommeliers with 10 controls, participants underwent a “blind taste test” during scanning (Pazart et al., 2014). During the tasting phase of this study there was enhanced activation for the sommeliers in the right anterior insula, as well as the hippocampus and regions in the occipital cortex. However, prior to tasting wines, sommeliers always use olfaction to make decisions and judgments about the wine that they are about to experience. Olfaction has been shown to be more consistently unilateral (right dominant) whereas gustation seems to involve both hemispheres (de Araujo et al., 2003). There have been no studies, to date, assessing the differences between activation patterns in sommeliers and those without special expertise in wine during the smelling of wine, despite this being a stage in the process when they make their initial and most complex judgments.

Studying experts in olfactory memory is important given that the regions involved in this task are frequently the first impacted by neurodegenerative disorders, including Alzheimer’s (Braak and Braak, 1995; Gomez-Isla et al., 1996) and Parkinson’s disease (Scatton et al., 1983; Braak et al., 2003). By studying structural and functional differences in experts who likely learned most of their specialized skill during adulthood, we will understand more about the plasticity of these regions important in aging, and perhaps inform future interventions.

This study examined whether structural brain differences were evident in sommeliers and if they correlated with accumulated years of experience. Such findings have been reported in other expertise groups (7). This correlation would be important since it would suggest a response to increased training and practice, as opposed to a pre-existing difference in their brains which may have predisposed sommeliers to becoming experts in their field. We also assessed differences between experts and controls during functional tasks associated with wine-judgments and compared these to visual judgment tasks, since we wanted to assess specific differences in the skill-set unique to sommeliers, not perceptual judgment more widely.

## Materials and Methods

### Ethics

All methodology was reviewed and approved by the Cleveland Clinic Institutional Review Board (IRB) for the imaging study. Pilot data used for stimuli-selection were collected at the University of Nevada, Las Vegas, under a protocol approved by their IRB.

### Participant Selection and Recruitment

Master Sommeliers were recruited with the assistance of JJ, Master Sommelier and extensively involved in both training of sommeliers and in the local community. After being approached informally by him and allowing their contact details to be shared, they were contacted by DB or SB to ascertain interest. All Master Sommeliers were considered eligible. The Court of Master Sommeliers provides a diploma to those who pass their four-stage examination process^2^. There are only 219 Master Sommeliers worldwide, all of whom have passed this process that takes several years. By including only individuals with this distinction, we could be assured that we were assessing true experts. However, this restriction did limit the number of sommeliers who we could include, and hence posed a limit to the potential sample size. We were able to recruit from the Las Vegas resort community and surrounding regions. Controls were recruited via word of mouth and advertising from among the local professional and academic community at the University of Nevada, Las Vegas. Controls were matched as closely as possible to sommeliers on age and gender. To assess the extent of wine knowledge in the controls especially, a brief, 10-item, multiple-choice wine quiz was developed to assess wine knowledge. Controls scoring 90% or higher at the screening phase were excluded from the study (*n* = 2) since they were likely to have a higher level of wine-related expertise than most of the non-sommelier population.

### Task Design

#### Stimuli Selection: Pilot Study

After Institutional Review Board (IRB) approval from the University of Nevada Las Vegas (UNLV), a preliminary selection of possible stimuli was conducted with UNLV students. Twelve male participants between the ages of 21–33 were recruited to participate. Following informed consent, participants were given the wine knowledge test. For this pilot study, only participants scoring below 70% were included. Eight non-wine blends were made by mixing varying amounts of vodka, cognac, Fusion brand verjus (a non-alcoholic grape juice made from the same grapes as many wines), fruit essences and in some cases water-soaked oak chips. Participants were blindfolded using a cloth sleep mask. The researcher held a glass jar containing either wine or a non-wine underneath the participant’s nose and instructed the participant to inhale through their nostrils. During the first task, participants were asked to tell the researcher if they smelled a wine or a non-wine.

During the second task, participants were asked to tell the researcher if they smelled a white or a red wine. Four white wines and four red wines were included. All jars during both tasks were chosen at random. Immediately following the olfaction tasks, participants were led to a computer and asked to rapidly categorize variably pixilated pictures of zebra patterns or fingerprints to allow us to match this visual task with the olfactory tasks on difficulty of identification.

Scores on the wine knowledge quiz ranged from 20 to 70% with a mean of 45%. Participants were able to distinguish between wines and non-wines with accuracy, ranging from 58 to 100%. Accuracy in distinguishing white wines from red wines varied from 25 to 91%. Accuracy at distinguishing between zebra patterns and fingerprints varied from 93 to 100%. Stimuli were selected based on participant accuracy. Specifically, stimuli from the bottom and top twenty percent of piloted stimuli were chosen. From this preliminary data, two white wines (2011 Walt Chardonnay and 2010 Trimbach Gewurztraminer), two red wines (2008 San Leonino Chianti Classico and 2010 Januik Cabernet Sauvignon), and three non-wine mixtures (a blend of white verjus with a small amount of vodka (either Tito’s or Belvedere), and some combination of fresh lemon juice and very dilute pear or apricot essence) were chosen. Wines were opened, and non-wines mixed fresh, for each imaging session, with a maximum of three participants being run in a session.

#### Imaging Study: Olfactory Apparatus

**FIGURE 1:**
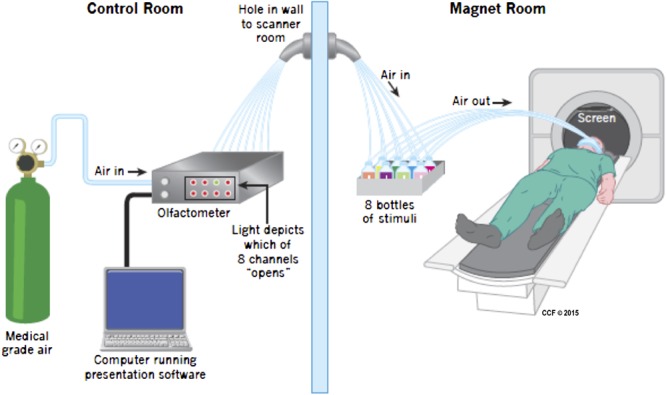
**Apparatus during scanning.** Medical grade air enters the olfactometer which controls release of air into one of eight channels, opened when instructed by the Presentation program. The air then traveled in a tube to one of eight bottles, seven of which included either wine or non-wine liquid, the other was empty. The air then went in a different tube to the mask attached to the face of the participant. The birdcage coil used during imaging is not depicted in the picture. Visual stimuli were presented using a mirror above the participant’s head.

Prior to entering the scanner subjects were fitted with a custom-designed mask consisting of a medical-grade oxygen face-mask modified to accommodate the placement of eight polyurethane tubes immediately below the nose. Each tube was attached to a small glass bottle containing a small amount (4 ml) of either wine (four bottles, two red and two white), or non-wine (three bottles), or nothing (one bottle). Additional tubing (polyurethane, 3.2 mm outer diameter and 1.7 mm inner diameter – Frethane 85, Freelin-wade, McMinnville, OR, USA) connected each vial to the olfactometer, located outside of the magnet room. The olfactometer is a computer controlled pneumatic stimulator (Institute for Biomagnetism and Biosignal analysis, University of Munster, Germany) that provides air pulses of well-defined duration. Its adaptation for olfactory research has been previously described (Frasnelli et al., 2010). The delay in getting the odorant from the trigger to the participant is 20–50 ms. The apparatus are depicted in **Figure 1**.

#### Tasks/Timing

##### Programming

**FIGURE 2:**
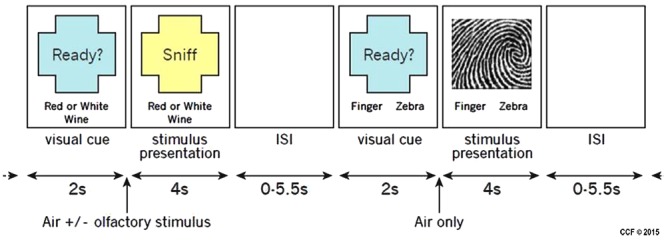
**Example of two stimulus presentations showing an olfactory stimulus (red or white wine) and a visual stimulus (fingerprint or zebra)**.

Stimuli were presented using a custom interface written in Presentation (Neurobehavioral Systems, Inc., Albany, CA, USA). Four different tasks, two olfactory and two non-olfactory, were presented pseudo-randomly in an event-related design. Each trial began with a 2-s visual cue informing participants which task to perform during the subsequent stimulus presentation which lasted 4 s. The inter-trial interval (ITI) varied from 0 to 5500 ms. For all tasks, participants reported their task-related decision using a two-button response pad. Button response-categories were reversed between participants so, for example, half the participants used the left button for red wine and half used the right button. The entire study consisted of two runs of 80 trials and lasted approximately 20 min. **Figure 2** illustrates the timing and tasks.

There were two different olfactory tasks. For both tasks, a single odorant was presented concurrently with an equal-duration visual cue that instructed participants to sniff, but also reminded them of the task to perform. Odorants were delivered for 4 s. During the red or white wine task, participants were pseudo-randomly presented air scented with one of two red wines or one of two white wines. Participants reported whether the odorant was from a red or white wine. A total of 40 trials were conducted, 10 of each odorant. During the wine or non-wine task, participants were pseudo-randomly presented odorants from a red wine, a white wine, or one of three non-wines. Participants reported whether the odorant was from a wine or a non-wine. A total of 40 trials were conducted, eight of each odorant.

There were two different non-olfactory tasks. During the fingerprint or zebra task, participants were randomly presented pixilated images of either a fingerprint or a zebra pattern (see **Figure 2** for example of stimuli). Participants were instructed to report whether the image was a fingerprint or a zebra pattern. A total of 40 trials were conducted, 20 of each image category. The image presented during each trial was unique so that no image was presented more than once. During the motor task, participants were delivered a 4-s stream of air through an olfactometer channel that contained no odorant and were instructed to respond by arbitrarily pressing the left or right button. A total of 40 trials were conducted. Due to a potential confound associated with the red or white wine task (the “clean” air pushed through during the motor task was potentially contaminated residual odor in the mask during the motor task, thus we had no adequate control) only the wine vs. non-wine olfactory task in comparison to the fingerprint vs. zebra visual control was analyzed in this pilot study.

#### Out-of-Scanner Tasks

General olfactory ability was evaluated using the University of Pennsylvania Smell Identification Test (UPSIT; Doty et al., 1984). The test consists of a booklet containing a series of standardized microencapsulated odorants. Scratching the paper releases each odor, which must then be identified by the participant (i.e., “scratch and sniff”). Results of this test indicate the degree to which individuals can identify smells in a forced-choice scenario.

Wine knowledge was assessed using the wine quiz. The wine quiz is a 10-item questionnaire developed for this project by MEP. The quiz contains questions about varietals, terroir, origin and tasting techniques.

### MRI Acquisition

All images were acquired on a Siemens Magentom Verio Syngo MR B17 3 Tesla MRI scanner with a birdcage head coil. A high resolution (1 mm × 1 mm × 1.2 mm) structural image was acquired using a 3-dimensional, T1-weighted gradient echo MP-RAGE sequence (repetition time = 2300 ms, Echo time = 2.98 ms, flip angle = 9). A total of 160 1.2 mm sagittal slices were acquired, each with a 256 × 256 matrix and 256 × 256 mm FOV, with parallel imaging (GRAPPA = 2, 24 reference lines). This was used in the VBM analysis.

Functional scans were acquired using gradient-echo T2-weighted echoplanar imaging (EPI), optimized for blood-oxygen-level-dependent (BOLD) contrast. Imaging parameters were TR/TE = 2500/28 ms; flip angle = 80°; field of view, 256 mm; slice thickness 4 mm; in-plane resolution/voxel size, 2 × 2 × 4 mm. A total of 240 volumes were obtained during each of two trial sessions.

### Data Analysis

#### Behavioral Data Analysis

Independent samples Student’s *t*-test were used to compare demographic details including Age and scores on the wine quiz and UPSIT tests. Repeated Measures ANOVA were used to compare accuracy and reaction time data between groups and between tasks. *Post hoc* testing was completed using the Least Square’s Differences approach. All data were analyzed using SPSS (v. 20, IBM).

#### Preprocessing of Structural MRI Data

Voxel-based morphometry (VBM) preprocessing was completed using SPM 8. The DARTEL tool was used to segment the structural image into gray matter and white matter, generate templates based on the scans used in this study, smooth to 8 mm and normalize these to MNI-defined standard space. We used the “preserve amount” option, thus utilizing modulation to reduce likelihood of error. VBM shows regions of relative density, and with the modulation option, volume, which differ between two groups.

#### Preprocessing of fMRI Data

fMRI data were analyzed using SPM 12. EPI data were input into a standard pre-processing pipeline that performed slice time correction, realignment, co-registration and normalization to the MNI 2 mm template. Finally, data were smoothed with an 8 mm Gaussian kernel.

#### Structural Data Analysis

Structural data were analyzed using a two-step approach. First, voxel-wise comparisons were completed using VBM in a region restricted to the frontal and temporal lobes since these covered the range of brain regions important in olfaction and memory where we suspected there would be a structural difference. This mask was made using the MARINA SPM extension. This allowed for the assessment of differences between sommeliers and controls in a large brain regions without very specific *a priori* hypotheses in an exploration unburdened by the restrictions imposed from corrections for multiple comparisons. Following preprocessing, MNI-normalized data were subjected to a two-sample *t*-test, comparing sommeliers and controls, with *p*_unc_ < 0.005 and a threshold voxel count of 50 and regions significantly different between the two groups were identified.

Secondly, we wanted to complete regional cortical thickness analyses based on the VBM findings. Freesurfer was used to calculate cortical thickness of parcellated regions of the entire brain for all subjects. Details on the usage of this software are documented elsewhere^3^. We visually checked for distortions. Cortical thickness values of individual regions of interest were extracted for each individual. We concentrated the analyses on those regions seen as significantly different on the VBM analyses. Cortical thickness of these regions were correlated with demographic variables, specifically age and years as a sommelier.

#### Functional Analysis

The pre-processed fMRI data were analyzed using SPM12. The first level design matrices of individual general linear models incorporated 11 regressors. Four regressors for the olfactory task (correct response to Wine-None-Wine task, correct response to Red-Or-White-Wine task, false response to Wine-None-Wine task, and false response to Red-Or-White-Wine task), three regressors for the non-olfactory tasks (correct response to Fingerprint-Zebra task, false response to Fingerprint-Zebra task, and response to Motor task), and four session related regressors (begin, instruction, fixation, and Mistrial time). Regressors were defined with onset of the event till the response of the participant. These 11 task based regressors were convolved with the canonical hemodynamic response function. At the second level, analysis was performed using ANOVA, assessing for group (sommelier vs. control), task [olfactory (wine/non-wine task) vs. visual (fingerprint/zebra task)], and interaction effects with age as a covariate. For this purpose, age was mean-centered by subtracting the overall mean age across all subjects. We used only correct responses to ensure that participants were attending to the task. The significance threshold for the group and task interaction results were determined using AlphaSim program (available in AFNI)^4^. The t-maps for the interaction were thresholded with an initial voxel-wise threshold *p* < 0.001 (uncorrected), and a cluster-extent threshold of *k* > 60 to achieve a cluster-level FWER corrected *p* < 0.05. We were particularly interested in the interaction, since we hypothesized that there would be a greater difference for sommeliers completing the olfactory task, compared with other effects. Following the ANOVA which was completed using SPM we extracted beta values in the regions involved in the interaction to visualize *post hoc* differences. The extracted Beta values from the relevant regions were also entered these into a correlation analysis with years of experience of the sommeliers.

## Results

### Participants and Behavioral Results

Thirteen individuals were recruited into each group, including two women in each group. Demographic characteristics and results of the wine quiz and UPSIT are summarized in **Table 1**. Sommeliers were, as expected, better on both the Wine Quiz and the UPSIT compared with controls. A normal score on the UPSIT is generally considered to be 34, so the sommeliers completed significantly better than normal, while controls performed as expected.

**Table 1:** Demographic, wine quiz and UPSIT scores for the two groups.

	Sommeliers(*n* = 13)	Control(*n* = 13)
Number of women	2	2
Age (Mean, *SD*)	44.42 (10.2)^∗∗^	34.00 (5.8)
Years as a master sommelier	8.92 (7.4)	Not applicable
Score on Wine Quiz (total/10 Mean, *SD*)	9.85 (0.4)^∗∗^	6.46 (1.8)
Score on UPSIT (total/40 Mean, *SD*)	36.8 (1.5)^∗^	34.23 (3.4)

*Sommeliers were older, and scored better overall on the wine quiz (^∗∗^*p* < 0.005) and UPSIT (^∗^*p* < 0.05) than the control group.*

During fMRI tasks, overall accuracy and reaction time were probed. Groups did not differ on accuracy or timing during any task, although there was a main effect of task on both these measures (accuracy [*F*(2,38) = 30.95, *p* < 0.0005] and response time [*F*(3,36) = 31.31, *p* < 0.0005]). This was driven by greater accuracy/faster response times during the visual task, which was completed with highest accuracy and quickest responses, compared to motor and olfactory tasks. There was also a significant difference between reaction time during the motor tasks compared to the visual and both olfactory tasks.

Only correct trials were used for the analyses. For the visual task these were on average 35.32/40 for sommeliers and 34.30/40 for the controls. For the wine non-wine task these were 27.69/40 for sommeliers and 26/40 for controls.

### Structural Differences

**FIGURE 3:**
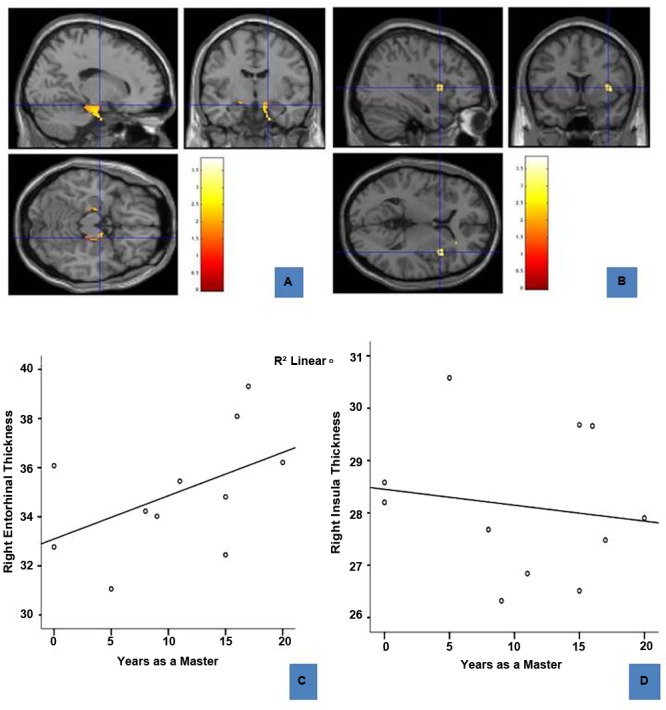
**Regions of enhanced volume in sommeliers and their correlation with experience: The entorhinal cortices (12, -8.5, -17.8) **(A)** and right insula (37, 12, 4.5) **(B)**, regions of greater volume in sommeliers compared with controls as depicted as an overlay on the MNI brain.** The thickness of the right entorhinal cortex is positively correlated with experience **(C)**, while the right insula thickness has no statistically significant relationship **(D)**.

Voxel-based morphometry results revealed three regions that differed between the two groups: sommeliers had higher volume in regions in the right and left entorhinal cortex, the right insula region, and in a small region in the left hippocampus (**Table 2, Figures 3A,B**).

**Table 2:** Regions that were significantly greater in volume for sommeliers compared with controls.

Region	Statistics			Coordinates (peak)		
	*k*	*t*	*p*	*x*	*y*	*z*
Right insula	162	3.85	<0.0005	37	12	4
Right Entorhinal	346	3.22	0.002	18	-10	-32
Left Entorhinal (extending to the left hippocampus)	166	2.78	0.005	-16	-27	-11

#### Relationships of Regional Cortical Thickness to Experience

To further explore any association between regional differences and expertise, right entorhinal and right insular thickness were obtained using Freesurfer and correlated with age and years as a master (i.e., years since passing the Master Sommelier exam). Results are shown in **Figures 3C,D**. There is a positive correlation between right entorhinal cortex thickness (*r* = 0.689, *p* = 0.047) and experience. There were no significant correlations for either the right insula (**Figure 3D**) or the left entorhinal cortex.

### Functional Differences

#### Task Based Differences

**FIGURE 4:**
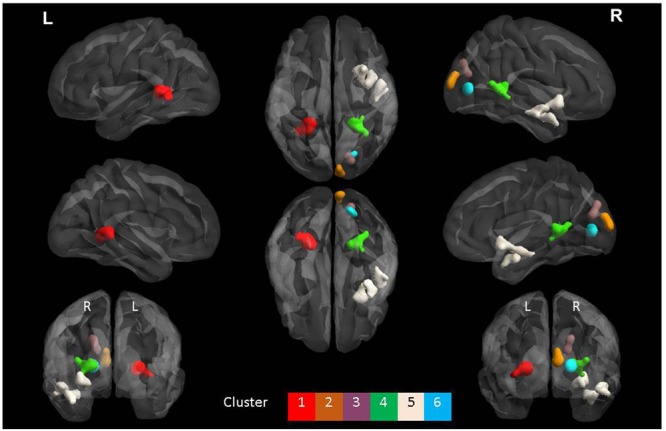
**The six clusters that were significant during interaction between group and task.** This figure was generated using the BrainNet viewer for a full list of all areas involved, see **Table 3**.

**FIGURE 5:**
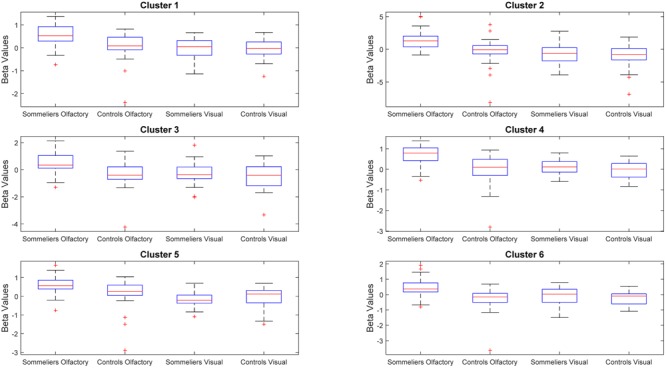
**Beta values within the clusters found to be significant during the interaction, for each group and each task**.

The ANOVA results revealed main effects for both group and task, and several regions of significant interaction between group and task. All results were adjusted for age which was included as a covariate of no interest, however age itself did not show any significant effect in the group or task comparisons, or interaction. For the main effect of group, Sommeliers showed five clusters as being more active, across tasks, compared with controls, these are detailed in **Table 3**. For the main effect of task, there were several very large clusters that were more active in the olfactory task, and very specific visual regions were more active during the visual task. These are also reported in **Table 3**. The regions involved in the interaction are detailed in **Table 4** and visualized in **Figure 4**. Most were in the right hemisphere, and in the regions around the olfactory cortices, limbic, and temporal memory regions, insula and some involvement of the visual cortex and association cortex. The one cluster in the left hemisphere was centered around hippocampus and visual cortices and a similar region in the right was also significant. Since this interaction was the central effect of interest, we used these regions to create a mask and extract mean Beta values for each group during task. The mean values and distribution of each of six significant clusters are graphically displayed in **Figure 5**. As expected, the sommeliers had a much higher level of activation than controls during the olfactory task. There were no apparent group differences on the visual task. Most clusters show highest levels of activation for the sommeliers during the olfactory task, and it is this finding that is likely to be driving the interaction effect.

**Table 3:** Results of main effects analyses.

Region	Number of voxels within cluster	Max *t*-value	Location(*x, y, z*)
**Main Effect of Group Somm>Control^∗^**			
Left Inferior Frontal Gyrus	66	4.667	-46, 20, 32
Right Middle Temporal Gyrus	142	4.295	44,-72,16
Right Insula	188	3.869	36,28,6
Left Inferior Occipital Gyrus	130	3.857	-30,-90,-4
Right Inferior Parietal Lobule	112	3.520	38,-52,50
**Main Effect of Group Control>Somm^∗^**			
None			
**Main effect of Task Visual>Olfactory^∗∗^**			
Right Middle Occipital Gyrus	185	9.934	32,-80,-6
Left Middle Occipital Gyrus	64	9.313	-2,-86,6
**Main Effect of Task Olfactory>Visual^∗∗^**			
Right Rolandic Operculum	1119	13.032	62,2,12
Left Rolandic Operculum	2966	12.016	-58,-12,16
Right Cuneus	1291	11.058	4,-90,28
Left Middle Temporal Gyrus	203	9.237	-60,-56,-12
Right Putamen	940	9.927	20,4,2
Right Precentral Gyrus	310	11.03	54,-4,40
Left Thalamus Left Pallidum	502	9.855	-8,-30,8
Right Middle Frontal Gyrus	101	9.142	42,52,28

*Results are depicted with the peak of each cluster, size of cluster, *t* statistic and coordinates. Analyses were run with the following thresholds (^∗^cluster FWER *p* < 0.05; ^∗∗^cluster FWER *p* < 0.05).*

**Table 4:** The regions within each of the 6 clusters that were significant in the interaction between group and task.

AAL regions	Number of voxels within the region	Max*t*-value	Location(*x, y, z*)
**Cluster 1**			
Left Hippocampus:	21	4.6876	-24, -38, 6
Left ParaHippocampal:	5	3.475	-20, -40, -2
Left Lingual:	38	4.3467	-24, -42, 0
Left Fusiform:	3	3.4999	-32, -50, -2
Left Precuneus:	36	4.4838	-20, -44, 4
**Cluster 2**			
Left Calcarine:	5	3.2989	4, -92, 12
Right Calcarine:	36	4.5871	10, -94, 12
Right Cuneus:	69	4.2401	10, -96, 10
**Cluster 3**			
Right Calcarine:	2	3.266	18, -76, 18
Right Cuneus:	28	3.5786	18, -78, 22
Right Superior Occipital:	35	3.748	20, -82, 32
**Cluster 4**			
Right Hippocampus:	24	4.105	26, -40, 4
Right Calcarine:	23	4.1707	24, -44, 6
Right Lingual:	1	3.196	18, -46, 4
Right Precuneus:	42	4.5825	24, -42, 6
**Cluster 5**			
Right Olfactory:	6	3.7369	28, 12, -12
Right Insula:	26	3.7499	30, 14, -12
Right Hippocampus:	3	3.4244	36, -4, -22
Right Amygdala:	7	3.5475	36, 2, -22
Right Fusiform:	1	3.188	44, -22, -16
Right Putamen:	19	3.7641	30, 8, -8
Right Superior Temporal:	25	3.7388	50, -4, -12
Right Superior Temporal Pole:	23	4.058	42, 10, -26
Right Middle Temporal:	77	4.0555	50, -4, -14
Right Middle Temporal Pole:	16	3.8193	44, 8, -26
Right Inferior Temporal:	2	3.3986	46, -18, -18
**Cluster 6**			
Right Calcarine:	82	5.4126	20, -78, 6
Right Lingual:	36	4.953	20, -76, 4

*The number of voxels within each region, as defined by the Automated Anatomical Labeling Atlas (AAL) is reported, along with the maximum intensity of the voxels in that region and the co-ordinates.*

#### Correlation with Experience

There were no significant findings when assessing correlation between beta values extracted from the regions discussed above and years of experience as a sommelier.

## Discussion

This pilot study revealed differences in both structure and function of sommeliers’ brains compared with controls. We found that the entorhinal cortex, a region relevant to olfactory processing, and more particularly to olfactory memory, was both relatively greater in volume in sommeliers compared with controls, and that it’s thickness (in the right hemisphere) was correlated with experience. Other regions that have been shown to be important in olfactory discrimination, specifically the insula (Rolls et al., 2003; Plailly et al., 2007), were also larger in sommeliers, although a correlation between thickness and experience was not found. Large regions, including parts of the brain responsible for olfactory processing, memory, and crossmodal processing, were more active during olfactory tasks compared with visual tasks in sommeliers than in controls. The regions that were more active overlapped with the regions that were found to be larger in volume, and were also more extensive in the right hemisphere.

### Enhanced Volume of the Entorhinal Cortex

Given the importance of the region to olfactory memory, this finding was expected. Sommeliers spend years learning about the olfactory qualities and other aspects of wine, and no doubt draw on that memory whenever they make a judgment about wine. We further found a relationship between years of experience and cortical thickness of the right entorhinal cortex. In many ways, this finding echoes that of Maguire et al. (2000) in taxi drivers who show enlargement of hippocampal regions with driving experience. It is also similar to the earlier finding of Delon-Martin et al. (2013) in perfumers, who showed increased size of the piriform cortex, a region of olfactory cortex directly adjacent to the entorhinal cortex. Furthermore, although we larger volume of this region bilaterally, compared with controls, the volume of the right hemisphere was larger than the left, consistent with early research on hemispheric lateralization of olfactory memory (Jones-Gotman and Zatorre, 1993). The entorhinal cortex may additionally be involved in more primary odor perception or identification tasks in addition (Wilson et al., 2014), which would also be highly relevant to a sommelier’s experience and skills. Given this region’s sensitivity to aging and neurodegenerative disease, it is especially interesting that we found this result comparing an older group of sommeliers with younger controls.

### Enhanced Volume of the Right Insula

The dorsal subregion of the insula was found to be larger in volume in sommeliers. This is thought to be due to the importance of this region in combining multisensory and higher order cognitive processes, activities that sommeliers practice throughout their training and work. The cortical thickness of this region had no relationship with experience, however, which is interesting in comparison to the finding with the entorhinal cortex. It may be that this region changes early in the training process and then plateaus, or that only part of this heterogeneous region responds to cross-modal sensory expertise. Longitudinal studies would be needed to further explore the relative impact of training on different regions. Further study regarding the particular role of subregions of the insula in cross-modal expertise would also be of interest.

### Enhanced Activation in Sommeliers Compared with Controls

Multiple regions showed an interaction between group and task, that appeared to be driven by more activity in sommeliers than controls, specifically during the olfactory judgment task. Importantly, task performance was similar between the two groups, making us more confident in the activation differences. We made the tasks similar enough to sommeliers’ work to be meaningful, but also wanted the tasks to require a similar amount of attention in both groups, thus made the tasks entirely novel (i.e., not discriminating types of wine, but rather wine from non-wine). These regions included olfactory, limbic, visual imagery, and multimodal regions. They are similar to those reported in an earlier study with fewer subjects comparing the taste and after-taste of wine and water in sommeliers compared with controls (Delon-Martin et al., 2013; Pazart et al., 2014). We assume that the enhanced, more widespread, activation in sommeliers implies more complex processing of the same information. Overall, there was somewhat more activation in the right hemisphere of sommeliers compared with controls. This is consistent with earlier studies that suggest right hemispheric dominance for olfaction and olfactory memory (Jones-Gotman and Zatorre, 1993). There were some left hemisphere differences in the interaction, always with analogous differences in the right hemisphere, specifically, in the hippocampus, lingual gyrus and precuneus. Previous fMRI studies of taste in sommeliers have also shown mixed lateralization: one study comparing tasting of wine vs. glucose showed more left insula activity compared with controls during the after-taste period (Castriota-Scanderbeg et al., 2005), while the study by Pazart et al. (2014) showed enhanced right but not left anterior insula activation during tasting but not during aftertaste.

The visual region enhancements both in the main effect of group (sommeliers more than controls) and in the interaction are interesting, this could be due to training of master sommeliers to use multiple senses while learning about wine, and to use imagery (e.g., of the fruit and vegetable section of a grocery store) when blind tasting^5^. This might explain the apparently enhanced activation of these regions in sommeliers during the olfactory task.

We did not demonstrate a difference in activation over the piriform [which we consider to be part of the olfactory cortex (Zatorre et al., 1992) although one cluster extended into the olfactory cortex though only by a few voxels.] in the interaction. Others have noted the inconsistency of imaging studies in olfaction to specifically demonstrate piriform involvement (Zald and Pardo, 2000) although an alternative explanation here might be that this primary olfactory cortex is equally activated by both groups.

From its comparison to activation during a complex visual discrimination task, our results support the specificity of the enhanced activation seen during the olfactory discrimination tasks, and particularly so in sommeliers. While the olfactory network enhancement makes intuitive sense, the result of the current study point to the specificity of this finding. Had we not included this visual control task, one could argue that the Master Sommeliers attended more to perceptual judgment tasks in general, and that this was not specific to tasks involving olfactory stimuli. Our use of a visual judgment task as a comparison provides evidence for the specificity of enhanced cortical activation during processes involving chemical senses in sommeliers.

There was no relationship between enhanced functional activation and years of experience. Future longitudinal studies will be important in learning more about the interrelationship between activation intensity and experience. There may be a causal relationship with those regions that show enhanced activity early in the training process showing enhanced volume or thickness over time. Similarly, we did not find any significant differences in certain parts of the olfactory network including the orbitofrontal cortex. The regions that were different were more specifically related to olfactory memory and cross modal integration, and it might be that these are the particular strengths that are enhanced in sommeliers, however, future research and direct comparison with other olfactory experts such as perfumers might be important to further disentangle the regions that could be specifically enhanced in one expert group over another.

The findings reported here were statistically robust, but it remains a pilot study, and repetition of the study with more participants will be important. This may allow the detection of correlations with experience that were not observed in the current functional dataset, although they were seen with the structural data. Matching subjects on age will be an important feature of these studies. Future research assessing the longitudinal impact of training on student sommeliers might reveal more about brain changes. Given the restricted number of Master Sommeliers, any further studies might be multi-site in nature, or centered around the timing of a wine event when multiple sommeliers are located near one center. It would also be of interest to compare experts with different interests directly, such as perfumer and wine experts, or with different sensory expertise, such as comparing sommeliers with visual experts such as those who detect fraudulent art work, or finger print recognition experts. This would allow us to learn more about the specificity of perceptual expertise. We also cannot make claims about the cellular level mechanisms of changes with experience. The development of new techniques in PET imaging, including imaging ligands that detect microglial processes, might be useful in understanding this process as suggested by the authors of a recent review (Zatorre et al., 2012). In the future, we would balance the number of wines and non-wines used, as this may have enhanced a novelty effect for the non-wines in the current study as participants become more habituated to the two white wines more quickly than the three non-wines. Similarly, in future studies we will test our participants’ memory, to see whether the enhanced thickness and connectivity of these regions is correlated with memory performance on neuropsychological tests.

This study identified enhanced structural and functional patterns in the olfactory network of sommeliers. These findings are consistent with the learning they undergo in achieving the status of Master Sommelier. Furthermore, the volume of a region of the brain involved in olfactory memory was associated with experience, suggesting that the continued training results in morphological changes of the brain. These results speak to the plasticity of the adult brain in response to sensory expertise. Future research into therapeutic sensory-cognitive training in individuals at risk from neurodegenerative diseases, such as Alzheimer’s or Parkinson’s, which impact the same regions of the limbic system and entorhinal cortex, might provide an important clinical application of these results.

## Author Contributions

SB and GL came up with the original idea and study design. SB, GL, DW, DB, MN, and MP designed the study and tasks. EB consulted on and set up MRI acquisition protocols. JF was our olfaction and olfactory MRI expert. JJ was our consultant master sommelier and was central in both design and stimuli selection. SB, DW, MN, XZ, KS, and DC analyzed the data. SB, KS, MP, DC, and GL wrote the manuscript. All authors read and contributed to edits on the manuscript.

## Conflict of Interest Statement

The authors declare that the research was conducted in the absence of any commercial or financial relationships that could be construed as a potential conflict of interest.

## Footnotes

1 https://www.mastersommeliers.org/resources

2 http://www.mastersommeliers.org

3 http://surfer.nmr.mgh.harvard.edu/

4 http://afni.nimh.nih.gov/afni/doc/manual/AlphaSim

5 http://www.wsj.com/articles/SB10001424127887323696404578300182010199640
